# Conservative treatment in adult patient with reimplanted anterior teeth after traumatic avulsion with extensive bone loss: an 8-year follow-up

**DOI:** 10.1590/2177-6709.26.1.e21bbo1

**Published:** 2021-03-10

**Authors:** Matheus Melo PITHON

**Affiliations:** 1 Universidade Estadual do Sudoeste da Bahia, Departamento de Saúde I (Jequié/BA, Brazil); 2 Universidade Federal do Rio de Janeiro, Programa de Pós-graduação em Odontopediatria e Ortodontia (Rio de Janeiro/RJ, Brazil)

**Keywords:** Conservative treatment, Tooth injuries, Periodontal diseases, Orthodontic anchorage procedures, Adult

## Abstract

**Introduction:**

Orthodontic treatment in patients with traumatized teeth is a condition that needs good planning in order to achieve satisfactory results.

**Objective:**

To discuss approaches to orthodontic treatment of malocclusions associated with trauma followed by avulsion of anterior teeth, reimplanted after a short period of time.

**Case report:**

The treatment started with the distalization of upper posterior teeth, with the aid of mini-implants and sliding jigs, followed by the inclusion of anterior teeth in the arch, followed by intrusion of these teeth.

**Results:**

With the treatment, improved mobility of the anterior teeth was achieved, with better insertion into bone tissue. The most important factor for satisfactory treatment and a good prognosis for avulsion is the time the tooth remains outside the socket. Orthodontic treatment in patients with traumatized teeth is not contraindicated; however, clinical and radiographic aspects must be considered.

**Conclusion:**

Among the feasible orthodontic treatment options, the conservative approach can be a very favorable treatment alternative.

## RESUMO

**Introdução:**

O tratamento ortodôntico em pacientes com dentes traumatizados é uma condição que necessita de um bom planejamento, a fim de se conseguir resultados satisfatórios.

**Objetivo:**

Discutir as abordagens de tratamento ortodôntico de más oclusões associadas ao trauma seguido de avulsão de dentes anteriores, reimplantados após curto espaço de tempo.

**Relato do Caso:**

O tratamento realizado iniciou-se com a distalização dos dentes posteriores superiores, com auxílio de mini-implantes e *sliding jigs*, seguida da inclusão dos dentes anteriores na arcada e intrusão desses dentes.

**Resultados:**

Com a realização do tratamento, conseguiu-se melhoria na mobilidade dos dentes anteriores, com inserção mais favorável no tecido ósseo. O fator mais importante para o tratamento satisfatório e um bom prognóstico da avulsão é o tempo em que o dente permanece fora do alvéolo. O tratamento ortodôntico em pacientes com dentes traumatizados não é contraindicado; porém, aspectos clínicos e radiográficos devem ser considerados.

**Conclusão:**

Entre as opções de tratamento ortodôntico factíveis, a abordagem conservadora pode ser uma opção de tratamento bastante favorável.

## INTRODUCTION

The number of adult patients who seek orthodontic treatment increases every day.^1^ Advancements in esthetics and comfort of orthodontic appliances has boosted the demand for this treatment.^2^ ^,^ ^3^ Adult patients have some peculiarities that are inherent to their past history that make their treatment unique.^4^ ^,^ ^5^ Missing teeth, periodontal disease, and traumatized teeth are not uncommon among these patients.^6^

Traumatic injuries to permanent incisors and their supporting structures constitute a true dental emergency and require immediate assessment and management.^7^ Among traumatic dental injuries, avulsion is one of the most severe, and its prognosis is closely related to the actions taken from immediately after avulsion to tooth reimplantation.^8^ ^,^ ^9^

Tooth reimplantation is considered a conservative treatment aimed at reinserting the avulsed tooth into the socket, but several factors should be taken into account in order to achieve an acceptable outcome.^10^ Time out of the socket is the most important factor for satisfactory treatment of avulsion and for a good prognosis; therefore, the tooth has to be reimplanted immediately, so that its functions can be preserved.^10^ ^,^ ^11^

Orthodontic treatment in patients with traumatized teeth is not contraindicated; however, clinical and radiographic examination of the repair and/or complications after the traumatic injury should be performed before treatment.^12^ ^,^ ^13^ Accordingly, the aim of the present study is to report a clinical case of an adult patient with generalized bone loss whose anterior teeth had been avulsed after a fall and reimplanted, with subsequent orthodontic tooth movement for Class II malocclusion correction. 

## DIAGNOSIS

**Figure 1:**
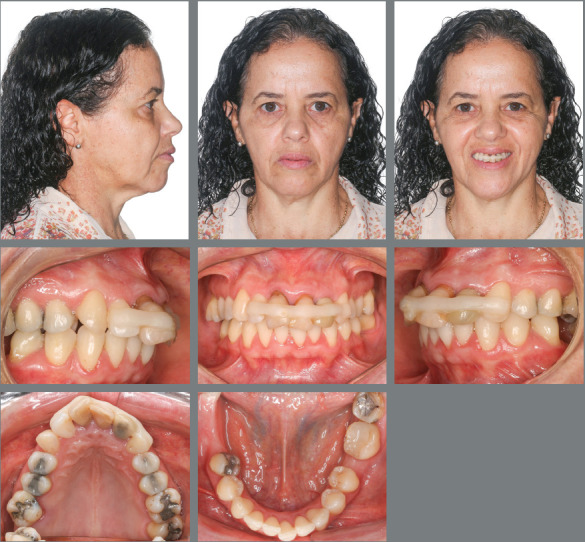
Initial extraoral and intraoral photographs.

Female patient, aged 49 years and 1 month, was referred by an implant dentist, who recommended malocclusion correction for later prosthetic rehabilitation of her missing posterior teeth and traumatized anterior teeth (Fig 1). The patient reported “*slipping in the shower about 10 months before, and feeling her anterior teeth falling right out of her mouth.”* The patient’s general health status was good, but her oral health was poor, since the following tooth elements were missing: #17 (maxillary right second molar), #36 (mandibular left first molar), and #46 and #47 (mandibular right first and second molars). The patient had extensively restored teeth and anterior teeth with large gingival recession, with history of trauma followed by avulsion of teeth #11, #21, and #22 (maxillary central incisors and maxillary lateral incisor). A full orthodontic workup was requested prior to the treatment.

**Figure 2:**
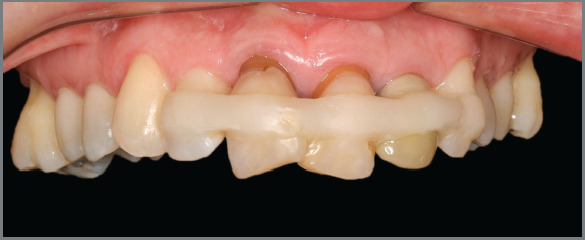
Initial frontal view of the maxillary dental arch, showing splinted and extruded reimplanted incisors, in addition to extrusion of maxillary right first molar.

The clinical extraoral examination revealed slightly enlarged lower third of the face, lip incompetence at rest, and dental protrusion. The intraoral examination showed Class II division 1 malocclusion, deep overbite (5 mm), overjet (5 mm), coincident midlines, maxillary and mandibular crowding (discrepancies of -4.3 mm and -3 mm, respectively), mandibular molars inclined mesially, extruded maxillary right first molar (#16) with gingival recession, in addition to extruded and splinted maxillary incisors with gingival recession (Figs 1 and 2). 

**Figure 3:**
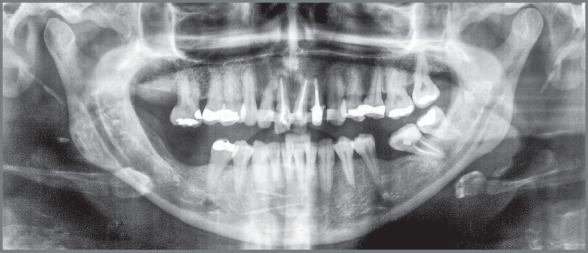
Initial panoramic radiograph.

**Figure 4:**
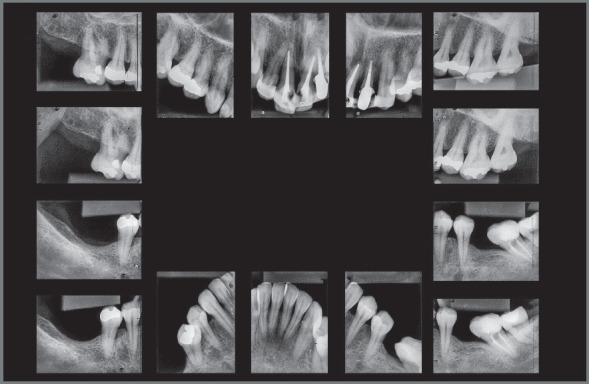
Initial periapical radiographs.

The radiographic examination revealed generalized bone loss, which was quite pronounced in maxillary incisors, maxillary right first molar (#16), and mandibular left second molar (#37). The anterior teeth with history of traumatic injury followed by avulsion (#11, #21, and #22) exhibited obturated root canals and largely restored crowns (Figs 3 and 4). There was also pronounced mesial inclination of mandibular left molars.

**Figure 5:**
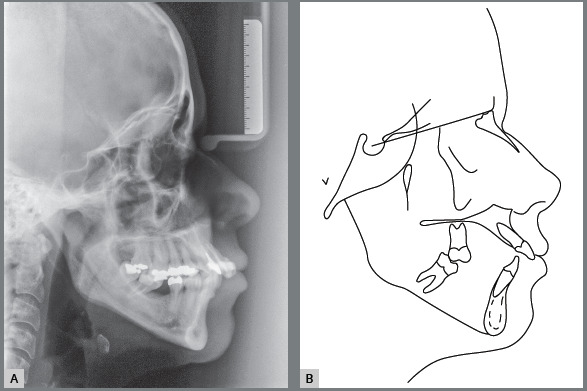
Initial cephalometric radiograph **(**A) and **(**B) cephalometric tracing.

The cephalometric analysis revealed skeletal Class II malocclusion (ANB = 5°), high mandibular plane angle (SnGoGn = 42°), and prominent and buccally inclined maxillary and mandibular incisors (1.NA = 39°, 1-NA = 10 mm, 1.NB = 33°, and 1-NB = 8 mm). The cephalometric profile indicated protrusion, with UL-S = +3 mm and LL-S = +4 mm (Fig 5). 

### TREATMENT OBJECTIVES

The treatment objectives of the present clinical case were as follows: creation of space in dental arches for orthodontic tooth movement; bilateral Class II relationship correction; bimaxillary protrusion correction; uprighting of mandibular molars, opening space for dental implants; intrusion and distalization of maxillary right first molar (#16); and maintenance and intrusion of traumatized maxillary incisors. 

### TREATMENT OPTIONS

» Extraction of maxillary central incisors (#11 and #21) and of maxillary left lateral incisor (#22), followed by their replacement with osseointegrated implants, in addition to distalization of maxillary posterior teeth supported by orthodontic mini-implants. » Extraction of maxillary central incisors (#11 and #21) and of maxillary left lateral incisor (#22), followed by mesialization of maxillary right lateral incisor (#12) towards the maxillary right central incisor (#11), as well as mesialization of maxillary right canine (#13) towards the lateral incisor. Placement of osseointegrated implants, with replacement of maxillary central incisor and reshaping of anterior teeth. » Conservative treatment with intrusion of maxillary central and lateral incisors, combined with Class II malocclusion correction with mini-implant-supported distalization of posterior teeth.

### TREATMENT PROGRESS

**Figure 6:**
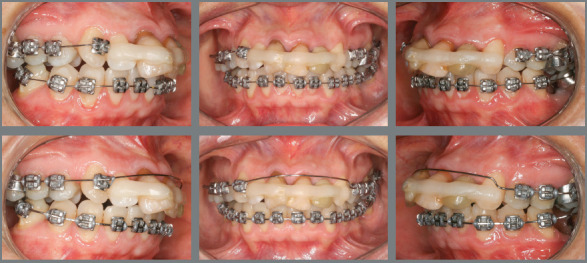
Tooth alignment and leveling at baseline. Maxillary archwire with anterior relief.

**Figure 7:**
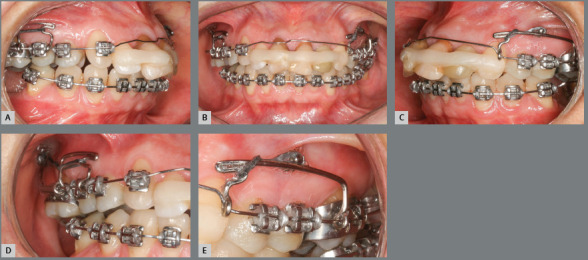
A-C) Initial distalization of maxillary teeth supported by mini-implants. D-E) Close-up views of the distalization system, consisting of sliding jig and mini-implant.

A conservative orthodontic treatment was proposed. Initially, the appliance was mounted with the Edgewise standard (0.022 x 0.030-in slot) continuous technique in the lower arch and segmented technique in the upper arch (Fig 6). Segmentation in the upper arch was aimed at aligning the maxillary posterior teeth, to allow future distalization with mini-implant-supported sliding jigs. Maxillary and mandibular alignment was made with 0.014-in, 0.016-in, and 0.018-in stainless steel archwires. After this, 0.020-in continuous archwires were placed for relief in the maxillary anterior region (Fig 7), whereas a 0.018 x 0.025-in archwire was placed in the lower arch. 

**Figure 8:**
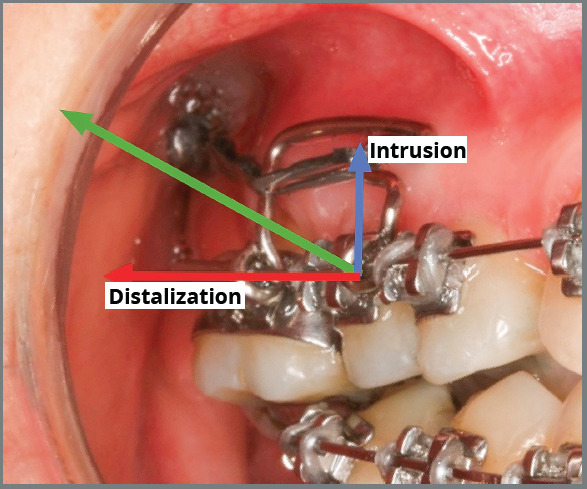
Distalization and intrusion of maxillary right first molar.

**Figure 9:**
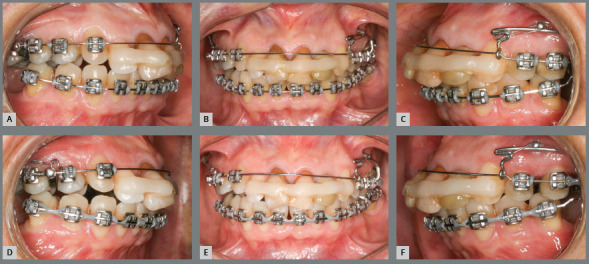
A-C) Molar distalization. D-F) Premolar distalization.

**Figure 10:**
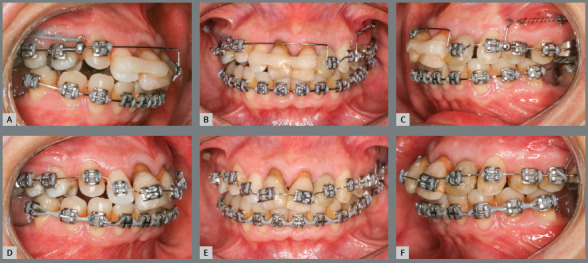
A-C) Premolar distalization with replacement of sliding jigs with archwire-supported hooks, with inclusion of tooth #22. D-F) Removal of splint, bonding of anterior teeth, and initial alignment and leveling.

After placement of the 0.020-in maxillary archwire, bilateral orthodontic mini-implants were inserted and used as support for the sliding jigs, for maxillary molar distalization. The mini-implant on the right side was positioned in the tuber region to create a resultant movement of intrusive and distalizing forces exerted on the maxillary first molar, which was extruded and mesially inclined. Premolar distalization was then obtained. A hook supported by a Gurin lock with an open coil spring on the right side and elastic chains on the left side was used (the sliding jig anchored the molars on the left side so that they would not move mesially during the insertion of the elastic chain between the molars and premolars) (Figs 8, 9 and 10).

**Figure 11:**
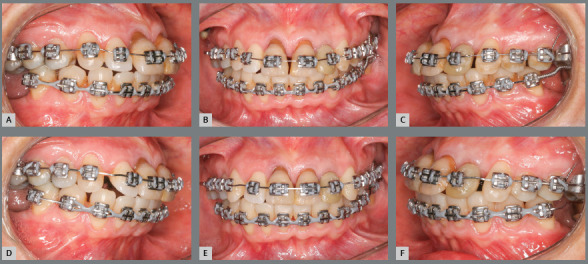
Anterior teeth in alignment and leveling stage.

**Figure 12:**
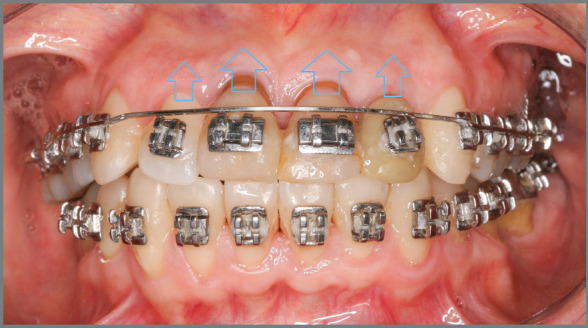
Activation of maxillary archwire, for intrusion of maxillary anterior teeth.

**Figure 13:**
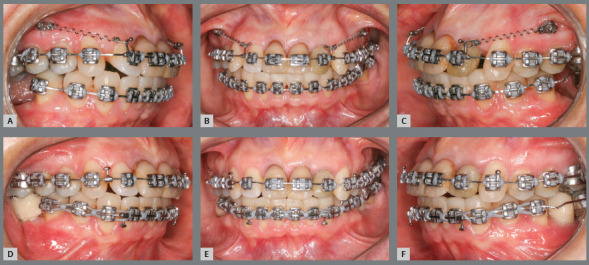
A-C) Placement of maxillary mini-implants, used for closure of maxillary spaces and intrusion of maxillary anterior teeth. D-F) Maxillary arch with closed spaces.

After creating a space in the maxillary left anterior region, the left lateral incisor (#22) was released from the splint and the orthodontic bracket was bonded. At this stage, another 0.020-in passive archwire was bent in this region and tooth #22 was included (Fig 11). This tooth had moderate mobility (grade 2). The splint was removed after 60 days and tooth movement was then checked. This stage of treatment caused a lot of concern, since the teeth had greater mobility. A 0.016-in passive archwire was chosen, since an active archwire could pull the teeth out. In subsequent visits, the archwire was adjusted as the teeth were aligned and leveled. After adjustment of the archwire and alignment and leveling of teeth, mobility was reduced. Because of that, 0.018-in, 0.020-in, 0.017 x 0.025-in, and 0.019 x 0.025-in archwires were used for tooth alignment and leveling. A step-up bend was used in the 0.019 x 0.025-in archwire, for intrusion of the extruded anterior teeth (Figs 12 and 13).

Meanwhile, in the lower dental arch, spaces were closed, tooth alignment and leveling were carefully adjusted, and the mesially inclined molars were uprighted. 

**Figure 14:**
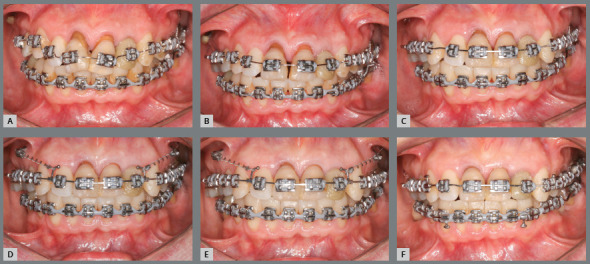
Frontal views during maxillary anterior teeth alignment and leveling associated with intrusion: A) Initial alignment and leveling; B) at 60 days; C) at 120 days, D) at 180 days; E) at 210 days, and E) at 270 days after the initial alignment.

**Figure 15:**
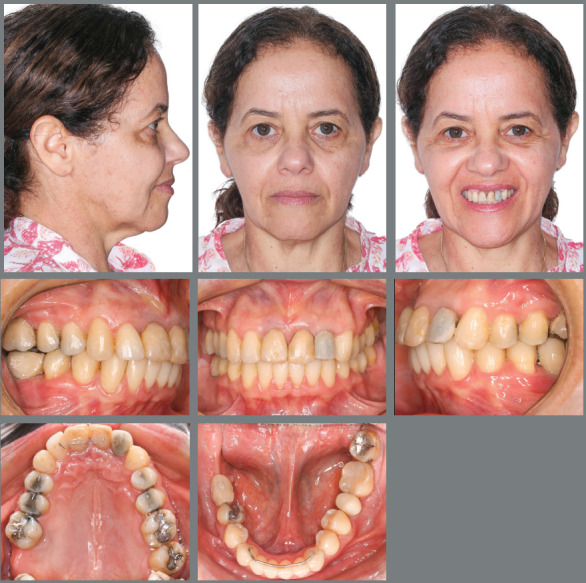
Extraoral and intraoral photographs at the end of treatment.

Thereafter, mini-implants were placed between maxillary premolars ##14/15 and #24/25, which served as support for closure of maxillary spaces, with posterior and maxillary repositioning (Fig 14). Note that, from the beginning of alignment and leveling, the anterior teeth became more intruded, improving their relationship with the other teeth and with the bone base (Fig 15).

After 36 months of treatment, the orthodontic appliance was removed, with subsequent placement of a 3x3 intercanine retainer. A wraparound retainer was used in the maxillary dental arch, in association with a fixed retainer between the maxillary central incisors and the maxillary right lateral incisor (#12 and #21) (Fig 15).

The patient was referred to a prosthetist for replacement of provisional prostheses on anterior teeth by definitive ones, in addition to periodontal follow-up. The prosthesis on the anterior teeth kept them together despite of extensive bone loss.

### TREATMENT RESULTS

**Figure 16:**
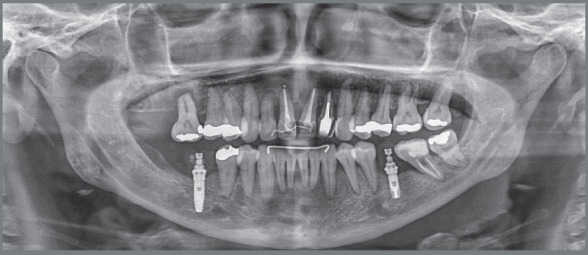
Panoramic radiograph at the end of treatment.

**Figure 17:**
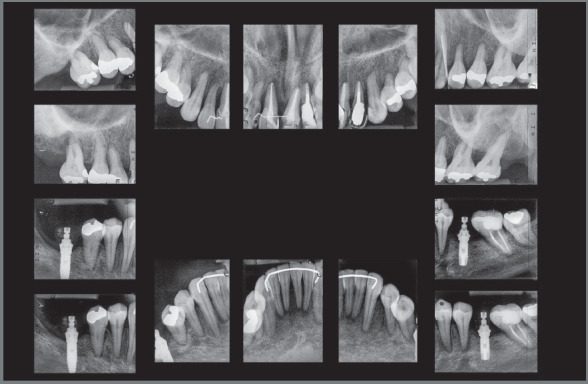
Periapical radiographs at the end of treatment.

At the end of the orthodontic treatment, there was enough space for accommodation of teeth, good intercuspation, with overbite and bilateral Class II malocclusion correction. The mandibular molars were uprighted, creating space for the placement of osseointegrated implants. Esthetically, smile harmony and positioning of the teeth were enhanced (Fig 15). The anterior teeth were intruded, substantially improving mobility, which went from grade 3, in central incisors and right lateral incisor, to grade 1 (Figs 16 and 17).

**Figure 18:**
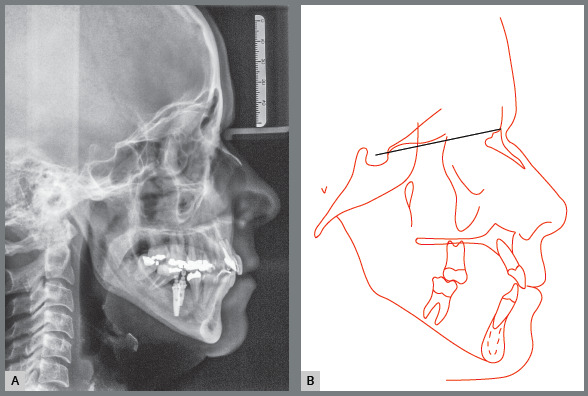
Cephalometric radiograph **(**A) and cephalometric tracing at the end of treatment **(**B).

**Figure 19:**
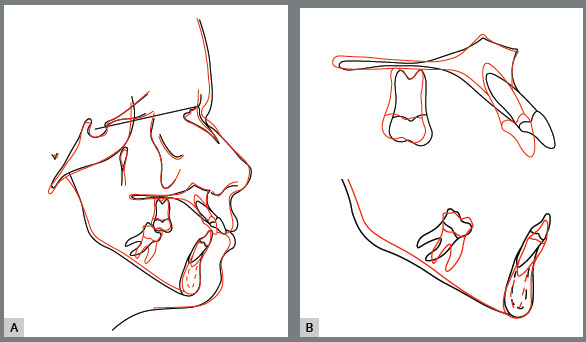
Total **(**A) and partial **(**B) superimpositions of the initial ( black ) and final ( red ) cephalometric tracings.

Radiographically, it was verified the intrusion of the maxillary right first molar and of the maxillary anterior teeth, leading to better insertion of incisors into the bone bases (Figs 16, 17, 18 and 19). The lateral view shows that lip protrusion was corrected, improving positioning of the teeth. The mandibular plane remained stable. 

According to the cephalometric analysis, central and lateral incisors were repositioned, showing better insertion in bone bases at the end of the treatment. There was a change in point A after repositioning of the incisors, with consequent skeletal Class II malocclusion correction (Figs 18 and 19).

**Figure 20:**
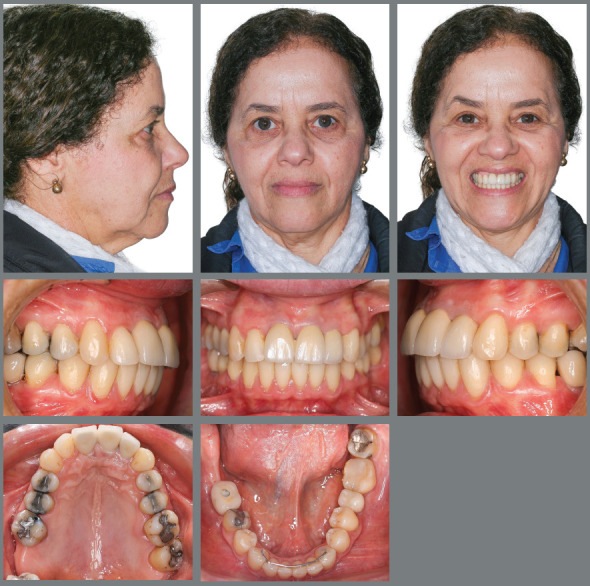
Extraoral and intraoral photographs 8 years after the end of treatment.

**Figure 21:**
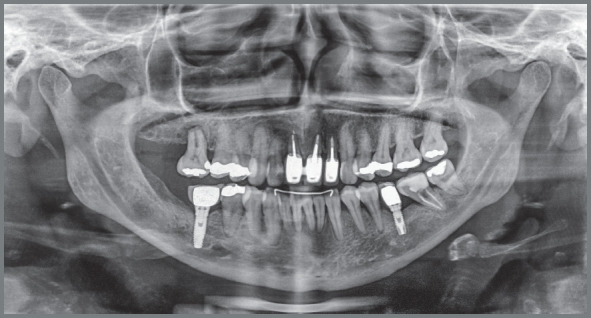
Panoramic radiograph 8 years after the end of treatment.

**Figure 22:**
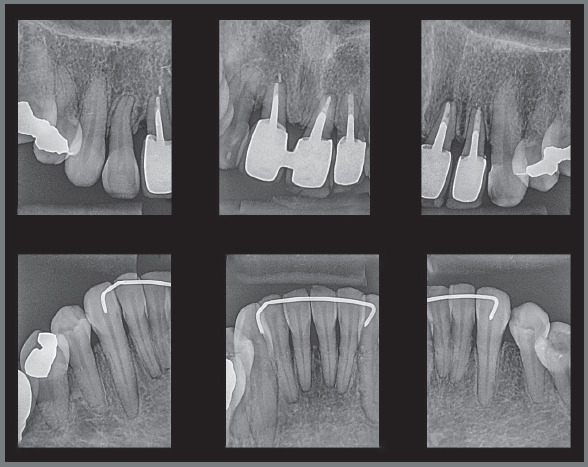
Periapical radiographs 8 years after the end of treatment.

**Figure 23:**
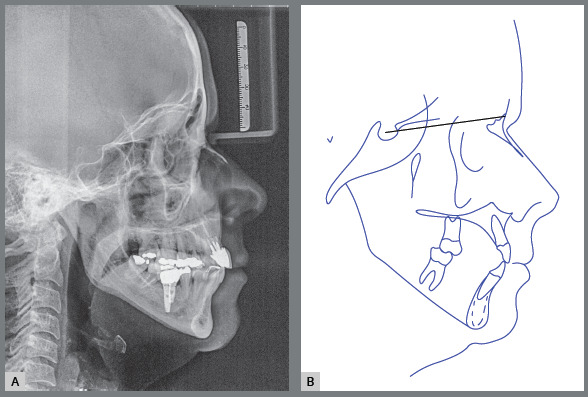
Cephalometric radiograph **(**A) and cephalometric tracing **(**B) 8 years after the end of treatment.

**Figure 24:**
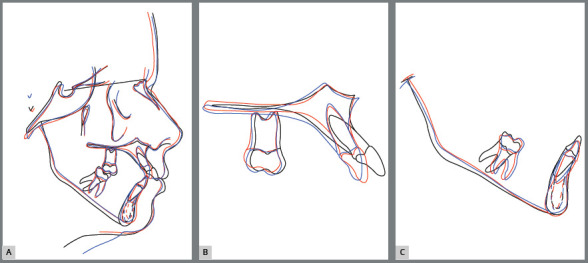
Total (**A**) and partial (B**-C**) superimpositions of cephalometric tracings: initial (black), final (red) and 8 years after the end of treatment (blue).

Eight years after removal of the fixed orthodontic appliance, the improvements achieved with its use were maintained (Fig 20), as it can be analyzed in the photographic and radiographic records (Figs 20 to 24 and Table 1).

**Table 1:** Initial (A), final (B) and 8-year follow-up (C) cephalometric values.

		MEASURES	Normal	A	B	Diff. A/B	C
Skeletal pattern	SNA	(Steiner)	82°	83°	84°	1	84°
	SNB	(Steiner)	80°	78°	80°	2	80°
	ANB	(Steiner)	2°	5°	4°	1	4°
	Angle of convexity	(Downs)	0°	9°	9°	0	9°
	Y-axis	(Downs)	59°	59°	58°	1	57°
	Facial Angle	(Downs)	87°	90°	91°	1	91°
	SN.GoGn	(Steiner)	32°	42°	42°	0	42°
	FMA	(Tweed)	25°	32°	28°	4	27°
Dental pattern	IMPA	(Tweed)	90°	93°	85°	8	87°
	1.NA (degrees)	(Steiner)	22°	39°	24°	15	17°
	1-NA (mm)	(Steiner)	4 mm	10mm	5mm	5	3mm
	1.NB (degrees)	(Steiner)	25°	33°	27°	6	28°
	1-NB (mm)	(Steiner)	4mm	8	7°	1	6°
	- Interincisal angle	(Downs)	130°	103°	125°	22	128°
	1 - APg	(Ricketts)	1mm	9mm	7mm	2	7mm
Profile	Upper Lip - Line S	(Steiner)	0	3	0	3	-0.5
	Lower Lip - Line S	(Steiner)	0	4	0	4	0

## DISCUSSION

The aim of the present study was to describe the conservative orthodontic treatment of a patient with Class II malocclusion whose anterior teeth had been reimplanted after their avulsion during a fall. The traumatic injury to the teeth might have been due to pronounced overjet (5 mm at baseline). According to Nguyen et al,^14^ individuals with an overjet greater than 3 mm have twice the risk for injury to their anterior teeth than those with an overjet of less than 3 mm.

Dental avulsion is characterized by total detachment of the tooth from its socket and accounts for approximately 0.5% to 16% of dentoalveolar injuries to permanent teeth.^15^ The time between avulsion and reimplantation, as well as where the tooth was kept during this period, are crucial for the prognosis, which is oftentimes poor. 

The patient described in this study reported that her teeth had been knocked out of her mouth and fallen to the floor after she had slipped in the shower. She also reported being unconscious for about 5 minutes and receiving first aid from a public emergency service before being taken to hospital, where a dentist reimplanted her teeth. On the way to the hospital, her teeth were kept in physiological saline, which is considered as the best transport medium.^16^ The time between the traumatic injury and tooth reimplantation was shorter than 1 hour, and her teeth was maintained in physiological saline throughout. As recommended by Donaldson and Kinirons,^17^ the teeth should not be allowed to become dry for longer than 15 minutes. Proper storage within a short period of time might have favored the good prognosis of her teeth.

Prior to the treatment, the patient had been referred to a periodontist for having her periodontal status checked and necessary procedures performed before orthodontic tooth movement. Note that if active gingival inflammation is controlled, intrusion can be a reliable therapeutic treatment in patients with reduced periodontal support, because it does not result in a decrease of the marginal bone level.^18^ ^,^ ^19^ For optimum results, intrusion should be performed with light forces, and the line of action of the force should pass close to the center of resistance.^20^ Light forces were used during intrusion. As mentioned earlier, alignment and leveling were performed with a passive stainless steel archwire, which was progressively adjusted as the teeth responded to orthodontic movement. After full alignment, orthodontic mini-implants were placed and used as support for space closure and intrusion. The mini-implants were placed between the premolars in a position that allowed the line of action of the force to be inclined upward, with anterior distalization and intrusion. The same rationale was applied for distalization and intrusion of the maxillary right first molar. In this case, the mini-implant was placed in the tuber, allowing the line of action of the force between the sliding jig and the mini-implant, to favor distalization and intrusion. Ahn et al.^21^ used a system with the same principle for the distalization and intrusion of protruded and extruded anterior teeth with periodontal loss. 

Before treatment, gingival recession of the anterior teeth was 2 mm, but it increased substantially during orthodontic treatment. Presumably, this occurred because of continuous extrusion of maxillary anterior teeth as an attempt to establish contact with the mandibular ones, and also because of the pronounced protrusion of those teeth.

Cardaropoli et al.^18^ evaluated the role of orthodontic intrusion in the reduction of gingival recession and probing depth around maxillary incisors of adult periodontal patients and found that the mean reductions in gingival recession were 0.96 and 1.71 mm at the buccal and mesial sites, respectively. In the present case, the reduction was more remarkable, as it was 5.5 mm at the beginning of intrusion of anterior teeth.

Nevins and Wise^22^ concluded that orthodontically moving teeth into infrabony defects might modify the defect’s morphology, reduce probing depth, and resolve the bony defect. This finding was described by Pithon^23^ for orthodontic movement of anterior teeth with extensive bone loss and also in the present clinical case. Better insertion was clinically evident, since in the new position the teeth showed lower mobility than at the beginning of orthodontic movement.

## CONCLUSIONS

The option of orthodontic treatment with a conservative approach can be a very favorable treatment alternative in malocclusions associated with trauma followed by avulsion of anterior teeth that are reimplanted after a short time.
